# Multimorbidity and polypharmacy in hospitalized older patients: a cross-sectional study

**DOI:** 10.1186/s12877-023-04109-4

**Published:** 2023-07-11

**Authors:** Yong Zhao, Jianchun Wang, Xiaojuan Zhu, Xiyu Zhang, Yahui Zhang, Wen Zhang, Yan Dong

**Affiliations:** 1 Department of Geriatric Cardiology, Shandong Provincial Hospital Affiliated to Shandong First Medical University, 324 JingWu Road, Jinan, 250021 Shandong China; 2 Department of Geriatric Hematology Oncology, Shandong Provincial Hospital Affiliated to Shandong First Medical University, Jinan, China; 3 Telemedicine center, Shandong Provincial Hospital Affiliated to Shandong First Medical University, Jinan, China; 4 Department of Pharmacy, Shandong Provincial Hospital Affiliated to Shandong First Medical University, Jinan, China; 5 Department of Geriatrics, Shandong Provincial Hospital Affiliated to Shandong First Medical University, Jinan, China

**Keywords:** Multimorbidity, Polypharmacy, Prevalence, Older patient, Hospitalization

## Abstract

**Background:**

The growing trend of ageing population has become a worldwide concern. In comparison with the youth, older people are more likely to suffer from multimorbidity and polypharmacy, both of which are associated with adverse outcomes and increased healthcare costs. This study aimed to investigate the status of multimorbidity and polypharmacy in a large sample of hospitalized older patients aged 60 years and over.

**Methods:**

A retrospective cross-sectional study was conducted among 46,799 eligible patients aged 60 years and over, who were hospitalized from January 1, 2021 to December 31, 2021. Multimorbidity was defined as the presence of 2 or more morbidities in one patient during the stay in hospital, and polypharmacy as prescription of 5 or more different oral medications. Spearman rank correlation analysis was used to assess the relationship of factors with the number of morbidities or oral medications. Odds ratio (OR) and 95% confidence interval (95% CI) were estimated from logistic regression models to determine the predictors for polypharmacy and all-cause death.

**Results:**

The prevalence of multimorbidity was 91.07% and increased with age. The prevalence of polypharmacy was 56.32%. Older age, polypharmacy, prolonged length of stay (LOS), higher cost on medications were significantly associated with an increased number of morbidities (all P < 0.01). The number of morbidities (OR = 1.29, 95% CI: 1.208–1.229) and LOS (OR = 1.171, 95% CI: 1.166–1.177) were potential risk factors for polypharmacy. As for all-cause death, age (OR = 1.107, 95% CI: 1.092–1.122), number of morbidities (OR = 1.495, 95% CI: 1.435–1.558) and LOS (OR = 1.020, 95% CI: 1.013–1.027) were the potential risk factors, but the number of medications (OR = 0.930, 95% CI: 0.907–0.952) and polypharmacy (OR = 0.764, 95% CI: 0.608–0.960) were associated with a reduction of mortality.

**Conclusion:**

Morbidities and LOS might be predictors for polypharmacy and all-cause death. The number of oral medications was inversely associated with the risk of all-cause mortality. Appropriate polypharmacy was beneficial for the clinical outcomes of older patients during hospitalization.

**Supplementary Information:**

The online version contains supplementary material available at 10.1186/s12877-023-04109-4.

## Background

The population in the world increased rapidly in recent decades, and it will reach 9.7 billion by 2050 [1]. Meanwhile, the growing trend of global ageing population has become a formidable problem. It is estimated that the size of older people aged 60 or over will be 2.1 billion in 2050 [2]. Older people are more likely to suffer from a broad spectrum of chronic disorders and use multiple medications than the youth. In most articles, multimorbidity was defined as the coexistence of 2 or more conditions in an individual [3]. Polypharmacy was regarded as the regular intake of 5 or more medications, and hyperpolypharmacy was described as clinical situations where more than 10 medications are taken [4]. Multimorbidity not only results in shorter life expectancy, worsening functional capacity, and poorer quality of life, but also is associated with longer hospital stays and increased healthcare costs [5]. Adverse outcomes of polypharmacy in older people include hospitalization and potentially inappropriate medications. However, the association between polypharmacy and mortality was conflicting [6]. In this report, we investigated the prevalence and associated factors of multimorbidity and polypharmacy, as well as the relationship of the number of medications with the risk of all-cause death, in hospitalized patients aged 60 years and over.

## Methods

### Study design and participants

A retrospective cross-sectional study was conducted by using a computer-assisted system. Older patients aged 60 years and over were included. They were hospitalized from January 1, 2021 to December 31, 2021, and their electronic medical records were reviewed.

This study was approved by the Ethics Committee of Shandong Provincial Hospital Affiliated to Shandong First Medical University (SWYX: NO. 2021 − 222). A waiver of informed consent from each patient was granted by the Ethics Committee of Shandong Provincial Hospital Affiliated to Shandong First Medical University due to anonymous use of the data for research purpose. All the procedures were in accordance with World Medical Association Declaration of Helsinki [7].

### Data collection

Demographic and medical information were collected. Patients were divided into 3 age groups: 60–69 years, 70–79 years, and ≥80 years. The main outcome measures were multimorbidity and polypharmacy, which were assessed according to the list of diagnoses at discharge and the number of oral medications documented in the medical records during hospitalization. In the present study, we considered multimorbidity as the presence of 2 or more morbidities in one patient during the stay in hospital based on the 10th International Statistical Classification of Diseases (ICD-10). These morbidities usually included, but were not limited to, chronic diseases, such as coronary artery disease, stroke, revascularization, hypertension, heart failure, dyslipidemia, arrhythmias, diabetes mellitus, and the like. Polypharmacy was defined as prescription of 5 or more different medications, and hyperpolypharmacy as 10 or more medications. In addition, laboratory results in the first few days of hospitalization were recorded.

### Statistical analysis

Continuous variables were expressed as mean ± standard deviation, and categorical variables as frequencies and percentages. Difference of continuous variables between groups was evaluated by one-way analysis of variance. Kruskal-Wallis test was used to determine the difference between medians of 3 or more independent groups. Categorical variables were compared by Chi-square test. In the multivariate analysis, Spearman rank correlation analysis was used to assess the relationship of factors with the number of morbidities or oral medications. Odds ratio (OR) and 95% confidence interval (95% CI) were estimated from logistic regression models to determine the predictors for polypharmacy and all-cause death. As for polypharmacy, age, gender, number of morbidities, and length of stay (LOS) were included. As for all-cause death, number of oral medications was included in addition to above potential factors. A two-sided P < 0.05 was considered to be statistically significant. All statistical analyses were conducted with Statistical Package for the Social Sciences software version 25.0 (IBM Corp., Armonk, NY).

## Results

### Characteristics of study patients

A total of 74,376 discharges were initially retrieved in this survey. For patients with 2 or more admissions in 2021, medical records of the first hospitalization were documented. Finally, 46,799 patients (56.35% males and 43.65% females) with an average age of 68.84 years were eligible for analysis. The proportion of patients aged 60–69 years, 70–79 years, and ≥80 years was 61.40%, 30.89%, and 7.71%, respectively. Table 1 shows the demographic and clinical characteristics of the study population.

**Table 1:** Demographic and clinical characteristics

			Age group (years)		P
	Overall	60–69	70–79	≥ 80	
Sample size	46,799	28,737	14,455	3607	
Age (years)	68.84 ± 6.57	64.60 ± 2.72	73.48 ± 2.72	83.94 ± 3.71	< 0.001
Male (%)	26,372 (56.35%)	16,037 (55.81%) ^a^	8316 (57.53%) ^a^	2019 (55.97%)	0.003
No. of morbidities	4.58 ± 2.52	4.34 ± 2.44 ^a, b^	4.86 ± 2.56 ^a, c^	5.36 ± 2.66 ^b, c^	< 0.001
No. of oral medications	6.01 ± 4.84	5.77 ± 4.62 ^a, b^	6.30 ± 4.99 ^a, c^	6.77 ± 5.77 ^b, c^	< 0.001
Length of stay (days)	8.09 ± 7.08	7.85 ± 6.20 ^a, b^	8.32 ± 7.51 ^a, c^	9.11 ± 10.76 ^b, c^	< 0.001
Overall hospitalization cost (CNY)	34518.05 ± 41088.73	34694.60 ± 39475.90^b^	35247.93 ± 42419.39^c^	30186.44 ± 47519.71 ^b,c^	< 0.001
Hospitalization cost per day (CNY)	4875.82 ± 4652.11	5002.15 ± 4573.28 ^a, b^	4827.28 ± 4882.65 ^a, c^	4063.95 ± 4224.00 ^b, c^	< 0.001
Overall medication cost (CNY)	5259.71 ± 9668.04	5127.16 ± 9009.88 ^a^	5480.29 ± 9879.64 ^a^	5431.74 ± 13211.41	0.001
Medication cost per day (CNY)	687.59 ± 983.28	693.96 ± 994.51 ^b^	693.37 ± 966.35 ^c^	613.75 ± 957.48 ^b, c^	< 0.001
All-cause death (%)	296 (0.63%)	86 (0.30%) ^a, b^	99 (0.68%) ^a, c^	111 (3.08%) ^b, c^	< 0.001
Hemoglobin (g/L)	130.76 ± 19.84	132.81 ± 19.19 ^a, b^	128.87 ± 19.97 ^a, c^	121.8 ± 21.21 ^b, c^	< 0.001
AST (U/L)	30.94 ± 130.62	30.39 ± 134.24	31.43 ± 123.08	33.36 ± 130.86	0.403
ALT (U/L)	26.06 ± 71.57	26.94 ± 73.25 ^a, b^	25.10 ± 72.56 ^a^	22.99 ± 50.59 ^b^	0.002
GGT (U/L)	44.86 ± 110.85	44.10 ± 103.94	45.71 ± 123.26	47.54 ± 111.47	0.134
Total bilirubin (µmol/L)	18.60 ± 33.54	17.98 ± 30.92 ^a, b^	19.12 ± 35.93 ^a, c^	21.44 ± 42.35 ^b, c^	< 0.001
Albumin (g/L)	38.71 ± 4.70	39.30 ± 4.54 ^a, b^	38.16 ± 4.69 ^a, c^	36.25 ± 4.87 ^b, c^	< 0.001
BUN (mmol/L)	5.95 ± 3.13	5.76 ± 2.87 ^a, b^	6.09 ± 3.24 ^a, c^	6.95 ± 4.26 ^b, c^	< 0.001
Creatinine (µmol/L)	71.38 ± 57.56	69.42 ± 59.25 ^a, b^	72.85 ± 52.55 ^a, c^	81.24 ± 61.81 ^b, c^	< 0.001
Cystatin C (mg/L)	1.06 ± 0.50	1.00 ± 0.49 ^a, b^	1.11 ± 0.49 ^a, c^	1.32 ± 0.58 ^b, c^	< 0.001
Potassium (mmol/L)	3.95 ± 0.47	3.96 ± 0.45	3.95 ± 0.48	3.95 ± 0.53	0.675
Sodium (mmol/L)	139.76 ± 3.34	140.01 ± 2.99 ^a, b^	139.56 ± 3.67 ^a, c^	138.57 ± 4.15 ^b, c^	< 0.001

ALT, alanine aminotransferase; AST, aspartate aminotransferase; BUN, blood urea nitrogen; CNY, China Yuan; GGT, gamma-glutamyl transpeptidase; SD, standard deviation

The superscripts, a, b, and c, indicate significant difference (P < 0.05) for the comparison between age groups 60–69 vs. 70–79, 60–69 vs. ≥80, and 70–79 vs. ≥80, respectively

The mean number of morbidities and oral medications increased with age. So did the prevalence of all-cause death. However, both the hospitalization cost per day and medication cost per day were significantly lower in the advanced ages (≥80 years) compared with those aged < 80 years. As for the laboratory findings, hemoglobin, hepatic function (assessed by total bilirubin and albumin), renal function (assessed by blood urea nitrogen, creatinine and cystatin C) and serum sodium were all decreased with age.

### Prevalence of multimorbidity

**Fig. 1:**
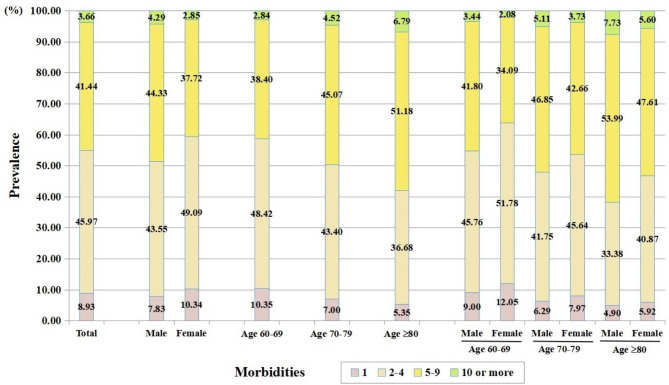
Proportion of patients with different number of morbidities by gender and age

As depicted in Fig. 1, the prevalence of multimorbidity (≥2 morbidities) was 91.07% for all patients. It was higher in males than in females (92.17% vs. 89.66%, P < 0.001) and increased with age (89.65% in age group 60–69 vs. 93.00% in age group 70–79 vs. 94.65% in age group ≥80, all P < 0.05).

If multimorbidity was defined as coexistence of at least 3 clinical conditions, the prevalence of multimorbidity would be 76.78% for all patients (79.14% for males and 73.73% for females). The estimate in the age group 60–69, 70–79, and ≥80 years would be 73.88%, 80.53%, and 84.84%, respectively.

### Prevalence of polypharmacy

**Fig. 2:**
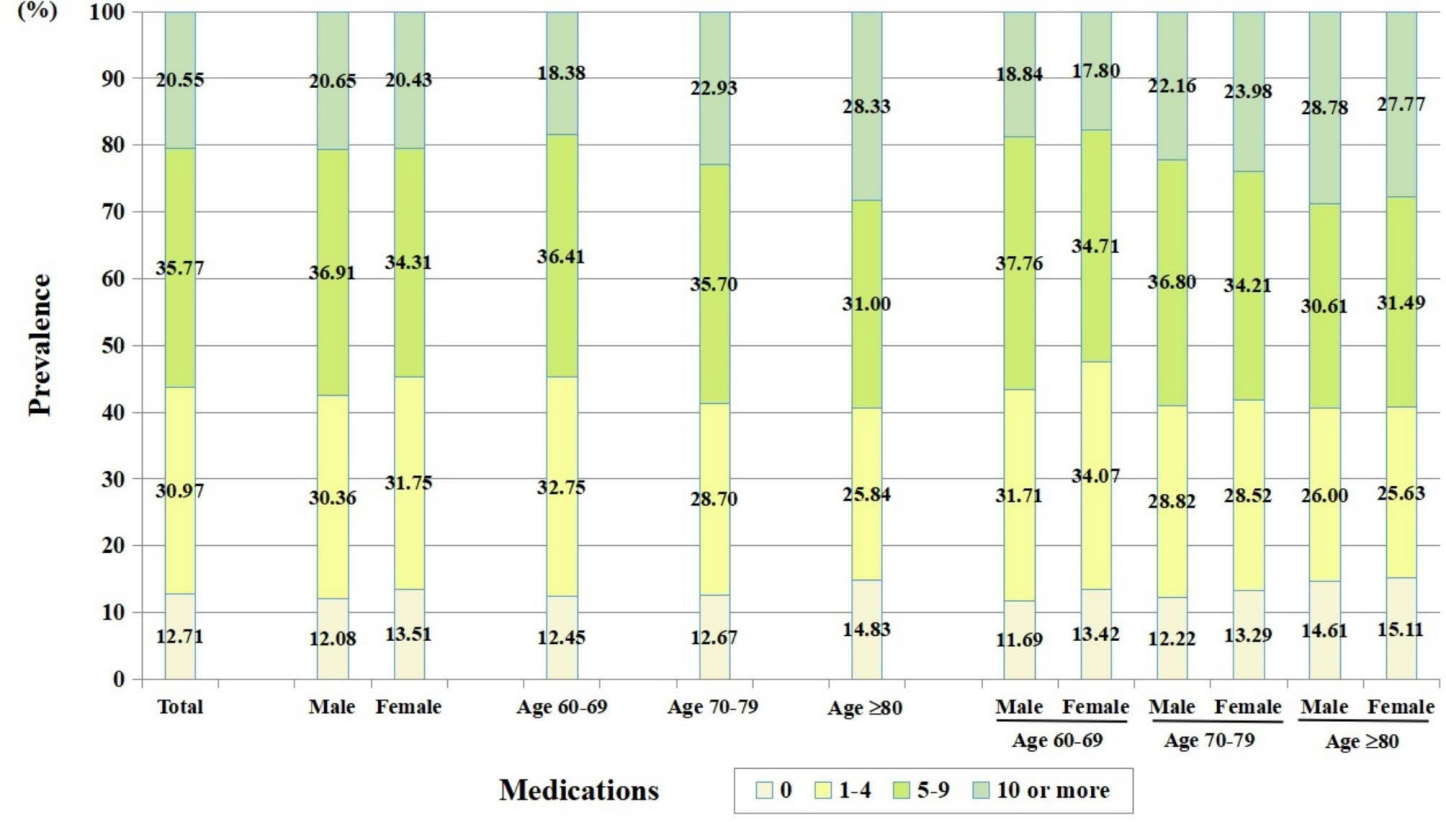
Proportion of patients with different number of medications by gender and age

As depicted in Fig. 2, the prevalence of polypharmacy (≥5 medications) was 56.32% for all patients. It was higher in males than in females (57.56% vs. 54.74%, P < 0.001). An increasing tendency with age was observed. The prevalence of polypharmacy was apparently lower in age group 60–69 (54.79%, P < 0.05), but similarly higher in age group 70–79 and age group ≥80 (58.63% vs. 59.33%, P > 0.05).

The prevalence of hyperpolypharmacy (≥10 medications) was 20.55% for all patients. It was similar between males and females (20.65% vs. 20.43%, P = 0.571), but markedly increased with age (18.38% in age group 60–69 vs. 22.93% in age group 70–79 vs. 28.33% in age group ≥80, all P < 0.05).

### Factors associated with multimorbidity, polypharmacy and all-cause death

Correlation analysis indicated that older age, polypharmacy, prolonged LOS, higher cost on medications were significantly associated with an increased number of morbidities (all P < 0.01). A positive relationship with the number of oral medications was found for age, number of morbidities, LOS, overall hospitalization cost and medication cost (all P < 0.01).

As shown in Table 2, the potential risk factors for polypharmacy were number of morbidities and LOS in hospital. As for all-cause death, age, number of morbidities and LOS were the potential risk factors, but the number of oral medications (OR = 0.930, 95% CI: 0.907–0.952) and polypharmacy (OR = 0.764, 95% CI: 0.608–0.960) were associated with a statistical reduction of mortality. Supplementary Figs. 1 and 2 show the dose-response relationship between numbers of morbidities (P < 0.001) or oral medications (P = 0.001) and mortality.

**Table 2:** Multivariable adjusted odds ratios for polypharmacy and all-cause death

	Polypharmacy				All-cause death		
Predictors	OR	95% CI	P		OR	95% CI	P
Age (years)	1.001	0.998–1.004	0.578		1.107	1.092–1.122	< 0.001
Gender (Female vs. Male)	1.018	0.978–1.060	0.389		0.874	0.683–1.117	0.282
No. of morbidities	1.219	1.208–1.229	< 0.001		1.495	1.435–1.558	< 0.001
Length of stay (days)	1.171	1.166–1.177	< 0.001		1.020	1.013–1.027	< 0.001
No. of oral medications	-	-	-		0.930	0.907–0.952	< 0.001
Polypharmacy	-	-	-		0.764	0.608–0.960	0.021

CI, confidence interval; OR, odds ratio

The cause of reduction in mortality risk of polypharmacy was examined in stratified analyses by age group and presence or absence of atherosclerotic cardiovascular diseases (ASCVDs). The odds of death for polypharmacy were 0.949 (95% CI: 0.621–1.450, P = 0.808), 0.748 (95% CI: 0.504–1.111, P = 0.151), and 0.551 (95% CI: 0.377–0.806, P = 0.002) in the age group of 60–69, 70–79, and ≥ 80 years, respectively. ASCVDs include coronary artery disease, stroke, peripheral artery disease, and other atherosclerotic diseases. The odds of death for polypharmacy were 0.364 (95% CI: 0.272–0.488, P < 0.001) and 0.962 (95% CI: 0.663–1.397, P = 0.840) in the patients with and without ASCVDs, respectively.

## Discussion

This large-sample retrospective cross-sectional study including 46,799 hospitalized patients aged 60 years and over provides considerable insight into the status of multimorbidity and polypharmacy in China. It showed that the mean number of morbidities was 4.58 and that of oral medications was 6.01 for a patient during the hospital stay. The prevalence of multimorbidity (≥2 morbidities) was 91.07% and that of polypharmacy (≥5 medications) was 56.32%. Both of them were higher in males than in females and increased with age. The number of morbidities was significantly associated with older age, polypharmacy, prolonged LOS, and higher cost on medications. The number of morbidities and LOS, but not age, might be risk factors for polypharmacy. As for all-cause death, age, number of morbidities and LOS were the potential risk factors, while polypharmacy was associated with a 23.6% reduction of mortality. These findings suggested that polypharmacy might be safe and beneficial for older patients during hospital stay.

Chronic diseases are the main cause of death for the global population, and older people are more likely to suffer from multiple disorders. In a cross-sectional study conducted in China [8], 258 oldest old hospitalized patients with polypharmacy were included. Their age ranged from 80 to 109 years. The number of chronic diseases was 3–13 per patient, with a mean of 7.0. In our study, the age of patients ranged from 60 to 104 years. The mean number of morbidities increased with age and it was 5.36 in the patients aged ≥80 years, which was lower than that in above study. This difference might be explained by the fact that the prevalence of polypharmacy was 59.33% in our patients aged ≥80 years but 100% in theirs, and polypharmacy was positively associated with the number of morbidities.

Multimorbidity was mostly defined as the presence of 2 or more chronic conditions, but a cutoff of 3 or 5 has also been used [9]. A scoping review on large database studies indicated that the prevalence of multimorbidity with a definition of at least 2 chronic conditions ranged from 15.3 to 93.1%, and that of multimorbidity with 3 or more chronic conditions varied from 11.8 to 89.7% [10]. A survey utilized the data of 19,841 participants aged ≥ 50 years from China Health and Retirement Longitudinal Study (CHARLS). Multimorbidity was defined as co-existence of 2 or more chronic diseases/conditions. The prevalence of multimorbidity was 42.4% for all participants [11]. As mentioned above, the prevalence of multimorbidity with ≥2 morbidities was 91.07% for our patients, which was remarkably higher than that among the participants from CHARLS. This difference might be due to the characteristics of subjects. On the one hand, the mean age of participants in CHARLS was lower than that of our patients (63.6 vs. 68.84 years). The prevalence of multimorbidity increased with age. On the other hand, CHARLS was conducted in community dwellers, and 29.2% of the participants did not have any of the 15 chronic diseases/conditions that were ascertained by the researchers. While our study focused on the hospitalized patients who had at least one disease, and a wider range of diseases were documented.

The prevalence of polypharmacy varied with the definition and population. A narrative review from Germany found that if polypharmacy was defined as ≥5 medications, the prevalence ranged from 4% among community-dwelling older people to over 96.5% in hospitalized patients [12]. While a cross-sectional study from Japan indicated that if polypharmacy was defined the current use of 6 or more medications, the prevalence would be 51.5% in the elderly home-care patients with an average age of 80.4 years [13]. In fact, identification of polypharmacy relies on not only drug count, but also a given time window. Cumulative polypharmacy, continuous polypharmacy, and simultaneous polypharmacy are 3 major types of polypharmacy indicators. Cumulative polypharmacy corresponds to the sum of all medications that were dispensed within a given time window [14]. A cross-sectional study in China by Chen et al. analyzed the prescription data from 720 hospitalized patients. Polypharmacy was defined as use of 5 or more medications in the last 3 months. Researchers found that the prevalence of polypharmacy was 51.12% in the patients aged ≥60 years, and polypharmacy was mostly a consequence of multiple chronic diseases [15]. In our study, polypharmacy was calculated as the cumulative use of 5 or more medications during the stay in hospital, and the cutoff for hyperpolypharmacy was ≥10 medications. We reported a similar prevalence of polypharmacy to the findings of Chen et al. in hospitalized patients.

A large sample cross-sectional study was carried out among 17,352 older patients aged 65 and over who were hospitalized in the geriatric department of 9 tertiary hospitals with multimorbidity and in China. The median number of medications used was 9, and 32.91% of patients were prescribed more than 10 medications [16]. Another retrospective single-center study in China reported that among 1,216 patients aged 65 years and over admitted in the geriatric department, 47.50% took 10 or more prescribed medications during hospitalization [17]. In the present study, we found that the prevalence of hyperpolypharmacy was 20.55% for all patients and 22.22% for those aged ≥65 years, which was notably lower than that in the patients of above two studies. Patients hospitalized in geriatric department might have more health problems than those in other departments, which might account for the difference in prevalence of hyperpolypharmacy.

Our findings revealed that polypharmacy was surprisingly associated with a reduction of all-cause mortality. Stratified analyses indicated that the risk was reduced predominantly in the patients aged ≥ 80 years and those with ASCVDs. However, a recent systematic review and meta-analysis on 24 studies reported that polypharmacy was associated with a significant increase in mortality in older adults with a pooled relative risk (RR) of 1.28 (95%CI: 1.19–1.39) [18]. Among the 24 studies, 6 were performed in hospital, but only 2 of them found this association, one in China and the other in the United States. In the Chinese study [19], a total of 1,562 participants were all male and aged ≥80 years. They were recruited at the geriatric outpatient clinic. In the American study [20], 324 patients aged > 65 years and with metabolic syndrome were followed up for 2 years at a tertiary medical center. Results showed that the number of medications ≤ 5 and > 10, but not 6–10, were associated with an increased risk of death. The ORs were 1.134 (95% CI: 1.001–1.152) and 1.153 (95% CI: 1.123–1.182), respectively [20]. The participants in above two studies were not hospitalized patients. The characteristics of them might be attributable to the difference in the findings from us. Any way, our study suggested that polypharmacy is not necessarily a risk factor for clinical outcomes. Rational use of multiple medications, especially under the supervision of clinical pharmacist, can be beneficial for older patients with serious or multiple morbidities. In other words, the quality of medications rather than the number should be the first priority for doctors to write a prescription.

The strength of this study is that the data were extracted from a large sample of patients hospitalized in a tertiary general hospital. The diagnoses covered a variety of clinical conditions and were confirmed by medical professionals. Prescriptions were written by medical practitioners and supervised by clinical pharmacists. Even so, several limitations have to be addressed. First, a cross-sectional design was used in this retrospective single-center study on hospitalized patients aged 60 years and over. The results cannot be extrapolated to other populations, such as community dwellers or individuals under the age of 60. Second, acute medical conditions were usually excluded from the definition of multimorbidity, because their impact on patients’ lives was not long-lasting and significant. However, they were included in the diagnoses of our study, which might be result in a higher prevalence of multimorbidity. Therefore, we also reported the estimate with the definition of multimorbidity as coexistence of 3 or more clinical conditions. Third, some oral medications taken by our patients, such as vitamins and single pill combinations (SPCs), were counted in the assessment of polypharmacy. Vitamins are not regarded as therapeutic medicines in most circumstances due to their little impact on clinical outcomes, and SPCs are composed of 2 or more pharmacologically active substances. Prescription of these medications might lead to a higher or lower prevalence of polypharmacy, Nevertheless, vitamins and SPCs accounted for a minimal proportion of the overall prescriptions in our patients and made little difference to the estimations. Fourth, the medications that should be administered parenterally have not been taken into account. Some of them are lifesaving chemicals, such as intravenous antibiotics or diuretics. Exclusion of these agents might underestimate the benefits of polypharmacy. Fifth, the association of following factors with polypharmacy or mortality should be examined, such as body mass index, frailty or activities of daily living, cognitive function, and/or socioeconomic status. Unfortunately, these data were not available in our study. Last but not least, although the association between polypharmacy and low mortality was derived from logistic regression in our study, it should be kept in mind that without adequate information on the appropriateness of prescriptions, mere quantity rather than quality of medications would overestimate the efficacy of polypharmacy.

## Conclusions

The prevalence of multimorbidity and polypharmacy were markedly high among hospitalized patients aged ≥60 years, especially for the male. The number of morbidities and LOS might be predictors for polypharmacy and all-cause death, while the number of oral medications and polypharmacy were associated with a reduction of mortality. Both the prevalence of multimorbidity and risk of death increased with age. Appropriate polypharmacy under the supervision of clinical pharmacist was safe and beneficial for the clinical outcomes of older patients during hospitalization.

## Electronic supplementary material

Below is the link to the electronic supplementary material.

Supplementary Material 1

Supplementary Material 2

## Data Availability

The datasets used and/or analyzed during the current study are available from the corresponding author on reasonable request.

## Abbreviations

ASCVD: Atherosclerotic cardiovascular disease
CHARLS: China Health and Retirement Longitudinal Study
CI: Confidence interval
LOS: Length of stay
OR: Odds ratio
RR: Relative risk
SD: Standard deviation
SPC: Single pill combination
