# Supplementary figures and images for: Multimorbidity and polypharmacy in hospitalized older patients: a cross-sectional study
Source: BMC Geriatr. 2023 Jul 11;23:423. doi: 10.1186/s12877-023-04109-4 (PMC10334650; doi:10.1186/s12877-023-04109-4)

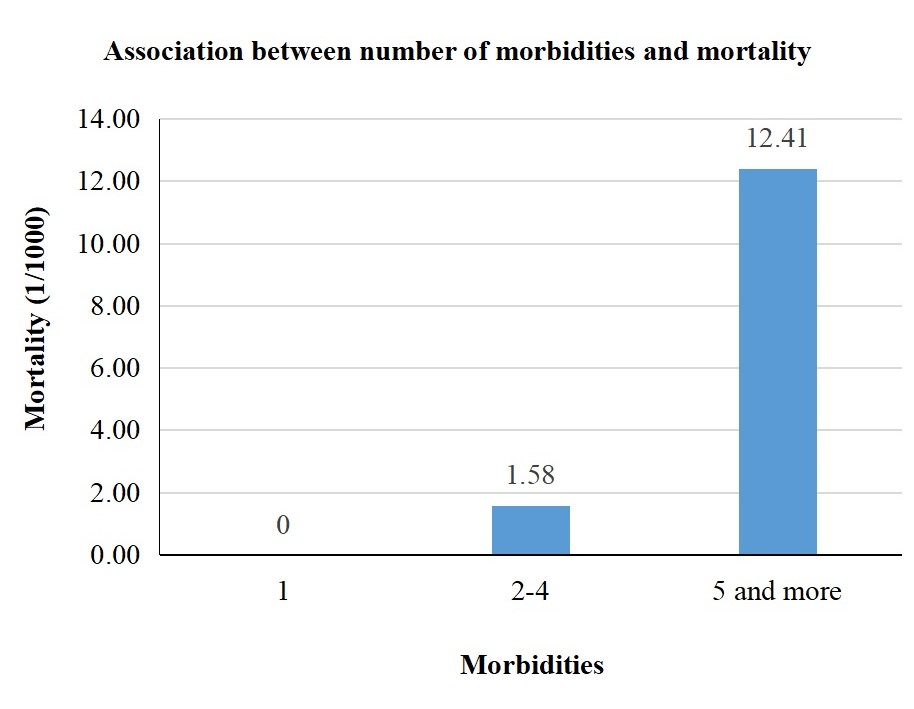

Supplement: Supplementary file 1 — Supplementary Material 1 [file 12877_2023_4109_MOESM1_ESM.jpg]

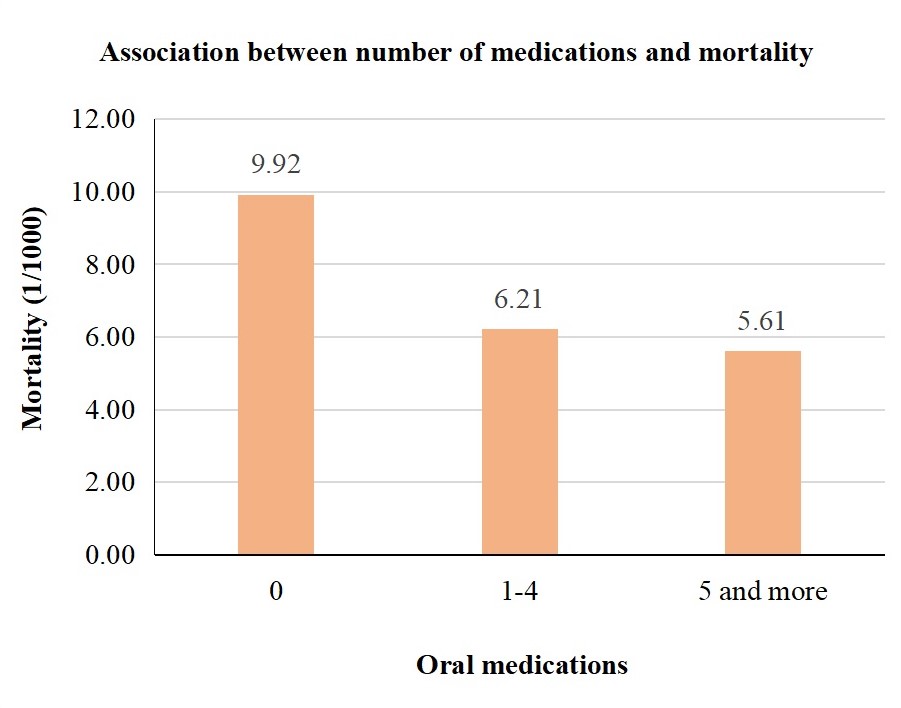

Supplement: Supplementary file 2 — Supplementary Material 2 [file 12877_2023_4109_MOESM2_ESM.jpg]
